# Health status of persons with dementia and caregivers’ burden during the second wave of COVID-19 pandemic: an Indian study

**DOI:** 10.1590/1980-5764-DN-2021-0100

**Published:** 2022-06-03

**Authors:** Ruchira Mukherjee, Bidisha Bhattacharyya, Adreesh Mukherjee, Goutam Das, Sujata Das, Atanu Biswas

**Affiliations:** 1 Bangur Institute of Neurosciences, Institute of Post Graduate Medical Education & Research, Department of Neurology, Kolkata, India.; 2 Rabindranath Tagore International Institute of Cardiac Sciences, Department of Neuropsychology, Kolkata, India.

**Keywords:** Caregiver Burden, COVID-19, Health Evaluation, Dementia, Fardo do Cuidador, COVID-19, Avaliação em Saúde, Demência

## Abstract

Due to the disruption of normal flow of treatment during the restrictions related to the coronavirus disease 2019 (COVID-19) pandemic, the health status of persons with dementia (PwD) and their caregivers’ burden might worsen.

**Objective:**

The article aims to find out the health status of PwD and caregivers’ burden during the peak of second wave of COVID-19 and make a comparison with the preceding trough phase.

**Methods:**

The study was conducted with 53 PwD and their caregivers in two phases. On their visit to the hospital during the unlock phase (phase 1), data were collected for CDR from PwD, and NPI-Q and ZBI from their caregivers. During the peak of second wave (phase 2), data were collected for NPI-Q, ZBI, and DASS-21 through telephonic communication, and statistical analyses were performed on the collected data.

**Results:**

Significantly higher caregiver burden (p=0.001) and neuropsychiatric symptoms (NPSs) [both in severity (p=0.019) and distress (p=0.013)] were observed among the respondents during the peak of second wave of the pandemic as compared to the preceding trough phase. Positive correlations were observed between the caregiver burden and depression, anxiety, and stress of the caregivers (p<0.001) and between the severity of dementia in PwD and caregiver burden (p<0.001) for both the first and second phases. Positive correlation was also observed between the severity of dementia in PwD and depression (p=0.042) and stress (p=0.023) of caregivers.

**Conclusions:**

Significant increase in the burden and distress was observed among caregivers due to increased NPSs of PwD during the second wave of COVID-19 pandemic.

## RESUMO.

Devido à interrupção do fluxo normal de tratamento durante as restrições relacionadas à pandemia de COVID-19, o estado de saúde das pessoas com demência (PcD) e a sobrecarga de seus cuidadores podem piorar.

**Objetivo:**

O artigo teve como objetivo conhecer o estado de saúde da PcD e a sobrecarga dos cuidadores durante o pico da 2ª onda de COVID-19 e fazer uma comparação com a fase anterior.

**Métodos:**

O estudo foi realizado com 53 PcD e seus cuidadores em duas fases. Em sua visita ao hospital durante a fase de desbloqueio (Fase 1), CDR, NPI-Q e ZBI foram administrados às PcD e seus cuidadores. Durante o pico da segunda onda (Fase 2), NPI-Q, ZBI e DASS-21 foram administrados por telefone e foram realizadas análises estatísticas dos dados coletados.

**Resultados:**

Foram observados sobrecarga do cuidador significativamente maior (p=0,001) e sintomas neuropsiquiátricos [tanto em gravidade (p=0,019) quanto angústia (p=0,013)] entre os entrevistados durante o pico da 2ª onda da pandemia em comparação com a fase anterior de passagem. Foram observadas correlações positivas entre sobrecarga do cuidador e depressão, ansiedade e estresse dos cuidadores (p<0,001) e entre gravidade da demência em PcD e sobrecarga do cuidador (p<0,001) tanto para a 1ª quanto para a 2ª fase. Também foi observada correlação positiva entre a gravidade da demência em PcD e depressão (p=0,042) e estresse (p=0,023) dos cuidadores.

**Conclusões:**

Foi observado um aumento significativo na sobrecarga e angústia entre os cuidadores devido ao aumento dos sintomas neuropsiquiátricos de PcD durante a 2ª onda da pandemia de COVID-19.

## INTRODUCTION

The coronavirus disease 2019 (COVID-19) pandemic has caused severe threats to public health both physically and mentally^1^. Across the world, the geriatric population being the most vulnerable group during the pandemic has faced its adverse effects^2^. The first severe acute respiratory syndrome coronavirus 2 (SARS-CoV-2) positive case in India was reported in the state of Kerala on January 30, 2020^3^. Thereafter, the number of cases started rising rapidly throughout the country, which was designated as the “first wave” of the pandemic. The peak of COVID-19 cases in the first wave in India was seen in September 2020^4^. To impose social distancing, a nationwide lockdown was initiated on March 25, 2020, and the same was extended in a phase-wise manner till May 31, 2020^5^. Subsequently, with reduction in number of cases in the country, the government announced resumption of services in phased manner termed as “unlock” period, which started on June 8, 2020^6^, and extended up to November 2020. COVID-19 cases once again started rising from March 2021, signaling the arrival of the second wave in India^7^.

During the spread of the pandemic, the number of lockdowns and unlock-downs were seen in India. ­Before the second wave entered the country, the trough phase of the disease was seen between December 2020 and February 2021 when there was a reduction in number of cases with easing of restriction called “unlock phase” with easier accessibility of resources. This allowed patients to avail consultations at hospitals and other health care facilities. Due to the second wave of COVID-19 in the country, partial lockdown was announced in different states.

In West Bengal, the peak of the second wave was seen between May and June 2021. Partial lockdown/self-imposed restrictions were announced in the state^8^. This included halting of rail and public transport services, limited hours for opening the markets, and night curfew, among others. Visiting health care facilities became difficult due to the lack of public transportation and fear of infection. The pandemic had its effects on daily living that were caused by shutting down of public venues, implementation of social distancing, economic downfall, and high levels of mortality across the population^9^ ^,^ ^10^ ^,^ ^11^.

Previous research on this global pandemic showed increase in mental distress^12^ ^,^ ^13^, especially in the vulnerable population like older adults^14^ and those in poverty^15^. One of the common diseases among older adults is dementia, which is associated with a greater risk of death^16^. The worsening of the disease is not solely due to vulnerability to infection^17^, but may also relate to the cognitive, behavioral, and psychological effects of rapid environmental changes brought by the pandemic. The vulnerability to the virus in patients with dementia is specifically related to their poor clinical status and their limited understanding of respiratory hygiene, such as hand sanitizing and the use of masks^18^. Deterioration of cognitive impairment in elderly persons with dementia (PwD) has also been reported following the pandemic^19^ ^,^ ^20^ ^,^ ^21^. Studies have also shown that community measures implemented to slow the spread of the virus have forced to social distancing and cancelation of cognitive stimulation programs, contributing to generate loneliness, behavioral symptoms, and worsening of cognition in patients with dementia^22^. Therefore, caregiver burnout is an expected consequence of increased demand for health care of PwD. Hence, the care provided by the caregivers may be troubled by their overwhelming load of work and homecare^23^.

Studies have described an abrupt worsening of neuropsychiatric symptoms (NPSs) of PwD, including depression, anxiety, aggression, agitation, and insomnia^24^, leading to an increase in distress among the caregivers^25^. Worsening of NPS leads to contamination^19^ and risk of self-injury, hospitalization, and death. Managing NPS in elderly PwD has been particularly challenging during the COVID-19 pandemic^19^. However, the effects of decline in the NPS and its burden over the caregivers are still unclear.

Informal caregivers of PwD experienced different difficulties during the pandemic that did not relate to their caregiving role^26^. Initially, hospital visits for regular follow-ups were difficult as well as the lack of certain necessary supply of goods and facilities followed by an overall drop in the economy^27^. In India, informal caregivers of PwD already face immense burden and stress due to the care they provide^28^. The lockdown followed by the first wave of pandemic caused incredible difficulties and challenges to PwD caregivers, increasing their caregiver burden^26^ ^,^ ^29^ and anxiety^30^. Despite a large number of PwD residing in low- and middle-income countries (LMICs) like India, studies on their health condition and caregiver’s distress during this pandemic are few.

Therefore, this study aimed to explore the change from the preceding unlock/trough phase during the pandemic in India, if any, in the burden of PwD caregivers and the patients’ health condition during the second wave of COVID-19.

## METHODS

The study was conducted with PwD and their caregivers. This is a part of an ongoing research of the department and permission was obtained for it from the Institutional Ethics Committee.

### Operational definitions

- *First phase of the study*: Unlock/trough phase between the months of December 2020 and February 2021 that had a decline in COVID-19 cases and relaxation of restrictions imposed by the government.
- *Second phase of the study*: Peak phase of COVID-19, i.e., second wave, in West Bengal, India, between May and June 2021.

### Sample

All PwD who visited our clinic during unlock/trough phase between the months of December 2020 and February 2021 were included in the study. PwD without a reliable caregiver was excluded.

### Procedure

Data were collected in two phases. In the first phase, data were collected for Clinical Dementia Rating Scale (CDR) from 54 PwD and CDR, Neuropsychiatric Inventory - Questionnaire (NPI-Q), and Zarit Burden Interview (ZBI) from their caregivers while they visited the clinic between December 2020 and February 2021, before the second wave hit India. In the second phase, i.e., during the partial lockdown when patients and caregivers were unable to visit the cognitive clinic of the hospital, data were collected for NPI-Q, ZBI, and Depression, Anxiety Stress Scale - 21 items (DASS-21) on the same caregivers of PwD through telephonic communication between May and June 2021. A psychologist (RM) collected the data in both phases. The caregivers were called and asked about their convenience of time and availability for the telephonic conversation. The purpose of the survey was explained to them, and the interview was conducted after their verbal approval. As one patient died due to COVID-19, the final sample consisted of 53 respondents.

### Tools

The following tools were used for the study:

- *Information Schedule* - A semi-structured questionnaire was constructed by experts, which included sociodemographic details along with the current COVID-19 and vaccination status of the patients and the caregivers. The patients’ health status was also included. Information were obtained from caregivers during the second phase of the study.
- *Zarit Burden Interview* (ZBI)^31^ - ZBI measures the subjective burden among caregivers of PwD and consists of 22 items rated on a 5-point Likert scale that ranges from 0 (never) to 4 (nearly always). The sum of the score ranges between 0 and 88. Higher scores indicate greater burden.
- *Neuropsychiatric Inventory - Questionnaire* ^32^ - This questionnaire provides a brief assessment of neuropsychiatric symptomatology of the patients and their caregivers’ distress related to it. It consists of 12 domains reflecting on the cardinal symptoms of the patient with responses “Yes” (present) or “No” (absent). In case of “Yes,” the informant is asked to rate the severity of the symptom on a 3-point scale and their own distress related to it on a 5-point scale. Total sum of the score in both 3- and 5-point scale reflects the severity and the distress related to it.
- *Depression, Anxiety Stress Scale - 21 Items* (DASS-21)^33^ - It is a scale that measures the emotional states like depression, anxiety, and stress. Each subscale contains 7 items and is rated on a 3-point scale ranging from 0 (not applicable) to 3 (very much). Summation of the score for each subscale reflects the severity of the emotional state from normal to severe. This scale was applied to caregivers of PwD.
- *Clinical Dementia Rating Scale* (CDR)^34^ - CDR is used to measure the severity of dementia. The global score is used for grouping patients on the severity of dementia in the categories of 0 (no impairment), 0.5 (questionable/very mild), 1 (mild), 2 (moderate), and 3 (severe). The sum of boxes is also used for grouping patients on the severity of dementia ranging from 0 to 18.00. In this study, the global scoring of the scale was calculated and used. While some responses of CDR were elicited from PwD, others were obtained from their caregivers.

### Statistical analysis

Statistics was carried out by using Statistical Package for the Social Sciences (SPSS version 21). Frequency (percentage) of categorical variables and mean (standard deviation) of the continuous variables were calculated. Pearson’s product moment correlation coefficient was used to analyze the significant relationship between ZBI and NPI-Q (both severity and distress) [first and second phases]; ZBI and DASS-21 (each subscale) [second phase]; CDR [first phase] and ZBI [first and second phases]; and DASS-21 (each subscale) [second phase]. Paired t-test was used to compare between first and second phase of ZBI and NPI-Q (both severity and distress). The p-value at the level of <0.05 was considered significant.

## RESULTS

### Demographic details

A total of 61 patients visited our clinic during the first phase of our study; of them, 54 were eligible for recruitment. As one of them succumbed due to COVID-19, a total 53 PwD were available for analysis. There were 32.1% female patients and 79.2% female caregivers in the sample. In all, 66.04 and 22.6% of patients and caregivers were of 60 years of age and above, respectively. The patients and the caregivers who had education till standard 10 and above were 58.5 and 69.8%, respectively. 3.8% patients and 24.5% caregivers were working. All were family caregivers providing informal care to PwD. Among them, 64.2% of caregivers were the spouse of the PwD and 32.08% were sole primary caregivers. 58.5% patients were suffering from the Alzheimer’s disease (Table 1).

**Table 1.** Characteristics of patients and primary caregivers.

Characteristics		Patient, n (%)	Primary caregiver, n (%)
Gender	Male	36 (67.9)	11 (20.8)
	Female	17 (32.1)	42 (79.2)
Age (years)	Below 60	18 (33.96)	41 (77.4)
	60 and above	35 (66.04)	12 (22.6)
Years of education	<10	22 (41.5)	16 (30.2)
	10 and more	31 (58.5)	37 (69.8)
Occupation	Working	2 (3.8)	13 (24.5)
	Non-working	51 (96.2)	40 (75.5)
Relation	Spousal	-	34 (64.2)
	Non-spousal (children)	-	19 (35.8)
Number of caregivers	Sole	-	17 (32.08)
	Multiple	-	36 (67.92)
Diagnosis	AD	31 (58.5)	-
	VAD	8 (15.1)	-
	FTD	7 (13.2)	-
	PDD	4 (7.5)	-
	Mixed	2 (3.8)	-
	DLB	1 (1.9)	-
Severity of dementia	Very mild	5 (9.43)	-
	Mild	15 (28.3)	-
	Moderate	18 (33.96)	-
	Severe	15 (28.3)	-
Vaccination status	Vaccinated	15 (28.3)	17 (32.1)
	Non- vaccinated	38 (71.7)	36 (67.9)
COVID-19 cases	Positive	4 (7.55)	4 (7.55)
DASS-21 (mild to extremely severe)	Depression	-	11 (20.8)
	Anxiety	-	11 (20.8)
	Stress	-	15 (28.3)

AD: Alzheimer’s disease; DASS-2: Depression Anxiety Stress Scale - 21 Items; DLB: dementia with Lewy bodies; FTD: frontotemporal dementia; Mixed: mixed dementia; PDD: Parkinson’s disease dementia; VaD: vascular dementia.

As diagnosed by CDR, 9.43% of the patients were suffering from very mild dementia, 28.3% each from mild and severe dementia, and 33.96% from moderate dementia. As reported by the participants, respectively, 28.3 and 32.1% of patients and carers were partially (single dose) vaccinated, and 7.55% COVID-19-positive cases each in patients and carers group who were found to have recovered (Table 1). Decline in patients’ memory was also reported by 47.16% of the caregivers.

### Caregiver distress

As calculated from DASS-21, 28.3% caregivers were found to suffer from stress and 20.8% each from depression and anxiety. Significant difference was found in caregiver burden (ZBI) and NPSs, both in severity and distress (NPI-Q) between the first and second phase of the data collection (Table 2). In ZBI, 26.42% caregivers reported financial difficulties in taking care of the PwD; 13.22% reported lack of socialization; 11.32% caregivers reported an increased feeling of stress between caring for the patient and trying to meet other responsibilities along with the fear of future regarding the patient; 9.43% reported anger, strain, and health deterioration due to the care they provide; and an equal number of caregivers also reported complete dependency of the patients on them.

**Table 2.** Difference in caregiver burden and neuropsychiatric symptoms between the first and second phases of the study.

	ZBI		NPI-Q			
			Severity		Distress	
Study phase	1st Phase	2nd Phase	1st Phase	2nd Phase	1st Phase	2nd Phase
Mean±SD	27.87±14.89	30.04±15.54	7.43±5	8.11±5	5.16±5.15	5.8±6
t-value	-3.58		-2.41		-3	
p-value	0.001*		0.019*		0.013*	

NPI-Q: The Neuropsychiatric Inventory; ZBI: The Zarit Burden Interview; *p<0.05 is considered significant.

As reported in ZBI, in the first phase, 18 participants had less or no caregiver burden, and in the second phase, there were 15 of them. Twenty-five participants reported mild-to-moderate caregiver burden in both the first phase and the second phase. Nine participants reported moderate-to-severe caregiver burden in the first phase and 11 reported the same in the second phase. One participant reported extremely severe burden in the first phase and two reported the same in the second phase.

### Correlates of caregiver distress

In between the first and second phase, a positive correlation was found between caregiver burden (ZBI) and NPSs, with both severity and distress (NPI-Q). Positive correlation was also found between second phase caregiver burden (ZBI) and depression, anxiety, and stress (DASS-21). A significant correlation (p<0.001) was also found between the severity of dementia (CDR) and both first and second phases caregiver burden (ZBI) along with depression and stress (Table 3).

**Table 3.** Correlates of caregiver burden.

Variables		Correlation (r)	p-value
NPI-Q and ZBI	Severity	0.912	<0.001*
	Distress	0.953	<0.001*
DASS-21 and ZBI	Depression	0.655	<0.001*
	Anxiety	0.491	<0.001*
	Stress	0.663	<0.001*
CDR and ZBI	First phase	0.333	0.015*
	Second phase	0.313	0.023*
CDR and DASS-21	Depression	0.281	0.042*
	Anxiety	0.197	0.157
	Stress	0.312	0.023*

CDR: clinical dementia rating scale; DASS-21: Depression Anxiety Stress Scale - 21 Items; NPI-Q: The Neuropsychiatric Inventory; ZBI: The Zarit Burden Interview; *p<0.05 is considered significant.

**Figure 1.**
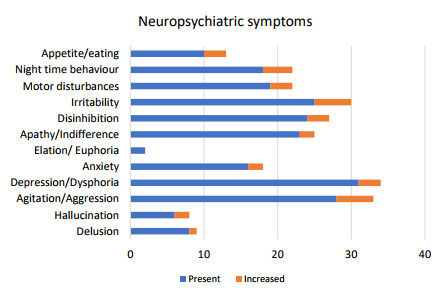
Frequencies of neuropsychiatric symptoms during the second wave of COVID-19.

As reported by the caregivers in NPI-Q, delusion, hallucination, agitation/aggression, depression/dysphoria, anxiety, apathy/indifference, disinhibition, irritability, motor disturbances, and problems related to eating were present in the first phase, which increased in the second phase (Figure 1).

## DISCUSSION

COVID-19 pandemic has affected the care of older adults with dementia severely^35^. The present study shows that there has been an increase in caregiver burden among informal carers of PwD during the second wave of pandemic. In this study, all were family caregivers and majority of them were spouse (64.2%) of the PwD. The depression, anxiety, and stress as well as burden of these caregivers should be viewed in relation with the bond and the time these caregivers spent with their near-and-dear one. The longing of these caregivers to keep their loved one safe and healthy with the limited resources during the pandemic increased the burden. Although this study did not attempt to compare the distress between family caregivers with professional one, literature say distress is much higher in the former^36^. Carers mostly reported difficulties regarding their financial condition and daily expenditure. This was probably due to the national economic and industrial downfall. They also reported lack of socialization due to stay-at-home order, fear of future uncertainty about themselves and the patients regarding the infection, and the fatality related to it. Difficulty in meeting family and work responsibilities along with caregiving, deterioration of their own health condition, and other psychological distress were also reported.

In an LMICs like India, which is among top five in COVID-19 cases till now, various concealed aspects of the pandemic have in one way, or another added to the difficulties of caregiving. Health care infrastructure, domestic issues, mental and physical health, and education system are challenged due to the lifestyle change. This is because of distant education, disrupted human resource management, effects on the labor class, monetary issues, lack of public transportation, unavailability for informal caregivers, etc., along with other difficulties faced by both the administration and the public during this pandemic^35^. Social distancing, stay-at-home order, and restrictions on gatherings, along with the unbalanced impact of COVID-19 itself on mortality and morbidity among older adults, have created challenges and changes to the type and intensity of caregiving, as well as to caregivers’ burden^29^.

The caregivers in this study mostly reported amplified NPSs like agitation/aggression, depression/dysphoria, anxiety, apathy/indifference, disinhibition, irritability, motor disturbances, and nighttime behavioral difficulties of PwD during the second phase. During COVID-19 second wave, NPS appeared to worsen after protracted isolation and lack of socialization due to environmental restrictions, which may have also cultivated behavioral disturbances.

Prolonged lack of proper medical follow-ups due to the pandemic may also lead to deterioration of health condition among PwD. This can lead to acute medical conditions, which might manifest increased NPSs like anxiety, agitation, and apathy^37^. However, as pointed out by Gilmore et al., emotional distress might also generate some NPS^38^. Social isolation and psychological symptoms may also increase cognitive (memory) decline in PwD during the pandemic^39^.

This study shows an increase in caregiver’s burden with increase in NPS and distress caused by it along with severity of PwD. This may be again due to the increased personal involvement of carers in terms of extensive amount of time for caregiving. Increase in NPS and severity of dementia can be attributed to the irregular medical follow-ups due to different restrictions during the pandemic leading to rapid deterioration of their health. Caregivers’ burden has been found to vary with the type of dementia due to varying pattern and severity of NPS in dementia subtypes^40^. However, in this study, we did not look into this.

This study also demonstrated that burden of caregiving increased with increasing severity of disease. The burden of caregiving inevitably increases with the progression of the disease^41^. Older adults with cognitive impairment are often taken care by informal caregivers, and the amount of this informal care is extensive and increases sharply as cognitive impairment worsens as pointed out by Langa et al.^42^. Prolonged period of the pandemic might also attribute to the negative apprehension of the carers regarding the patients’ health conditions. This increased burden may sometimes lead to psychological distress like stress and depression among the caregivers as seen in this study.

The limitation of the study was that the mode of data collection differed in the two phases: in phase 1, it was face to face; in phase 2, it was telephonic as the participants were not available for face-to-face interaction. Another limitation was the lack of previous data on depression, anxiety, and stress of caregivers to compare with that during the second wave of COVID-19. The strength of the study, however, is the availability of baseline data for CDR, NPI-Q, and ZBI obtained face to face during the unlock/trough phase preceding the second wave, which could be compared with the changes during the second wave.

In conclusion, this study shows significant increase in caregivers’ burden and distress among caregivers due to amplified NPSs of PwD in the second wave of COVID-19 pandemic. A positive correlation was also seen between caregiver burden and NPSs, regarding both severity and distress. Caregiver burden in the second phase was associated with depression, anxiety, and stress. Severity of dementia was also seen to be associated with caregiver burden, along with stress and depression among carers. Although our study clearly established increase caregivers’ burden in the second wave of COVID-19 and we could demonstrate its relationship with certain factors, some other factors not considered may also be related to caregiver stress.
